# Exploratory real-world experience with GLP-1 receptor agonists vs. metformin in youth with new-onset type 2 diabetes: a single-center retrospective study

**DOI:** 10.1515/jpem-2025-0493

**Published:** 2025-11-10

**Authors:** Isaac Tejeji, Troy Zeier, Josephine A. Smith, Nancy T. Chang, Lily C. Chao

**Affiliations:** The Center for Endocrinology, Diabetes, and Metabolism, Children’s Hospital Los Angeles, Los Angeles, CA, USA; Institute for Nursing and Interprofessional Research, Children’s Hospital Los Angeles, Los Angeles, CA, USA; Keck School of Medicine, University of Southern California, Los Angeles, CA, USA

**Keywords:** GLP1, type 2 diabetes, pediatric diabetes, metformin, obesity and type 2 diabetes

## Abstract

**Objectives:**

In youth with type 2 diabetes (YT2D), glucagon-like peptide 1 receptor agonists (GLP1) are recommended as adjuncts to metformin (Met) when glycemic targets are not achieved. Early GLP1 use may improve weight and glycemic control, but its efficacy as monotherapy in treatment-naïve YT2D remains unstudied. This exploratory study compares GLP1 and Met monotherapy for glycemic and weight outcomes in newly diagnosed YT2D.

**Methods:**

This retrospective study analyzed patients<21 diagnosed with T2D between January 2022 and March 2024 at a single center. Data were collected for up to 1 year following diagnosis for patients prescribed GLP1 or Met alone. Records were excluded if additional diabetes medication or bariatric surgery was introduced. A mixed effects linear regression model adjusted for baseline BMI, HbA_1c_, age, and gender.

**Results:**

The cohort included 12 GLP1 and 113 Met patients. About 83 % GLP1 patients were female (vs. Met 51 %). All were publicly insured. Median age at diagnosis was 14.8 years. Baseline and final HbA_1c_ were similar: GLP1 (52–36 mmol/mol) and Met (52–44 mmol/mol), with 83 % GLP1 and 67 % Met patients achieving HbA_1c_≤48 mmol/mol (6.5 %) (p=0.253). Baseline BMI was higher in GLP1 (46.37 vs. Met 35.06 kg/m^2^). Percentage BMI reduction favored GLP1 (−5.10 %) over Met (−0.59 %). Regression analysis showed GLP1 was associated with greater monthly percentage BMI reduction (β= −1.08 %, p=0.001) but not with HbA_1c_ change (β= −1.1, p=0.308).

**Conclusions:**

GLP1 led to greater BMI reduction with comparable glycemic control relative to Met in newly diagnosed YT2D.

## Introduction

The prevalence of obesity among adolescents in the United States continues to rise, with rates increasing from 17.7 % in 2011 to 21.5 % in 2020 [1]. The rise in obesity is accompanied by an increased incidence of type 2 diabetes, driven by obesity-related insulin resistance and β-cell dysfunction [2], 3]. Youth-onset type 2 diabetes is aggressive, with diabetes complications occurring in early adulthood [4], 5]. Weight reduction is associated with improvement in HbA_1c_ and other cardiovascular risk factors [6], 7]. Effective medications that target both weight and glycemia have the potential to reduce the effects of future type 2 diabetes complications. Currently, metformin remains the first-line medication for newly diagnosed youth-onset type 2 diabetes [8]. While it effectively reduces HbA_1c_ with initial use, the TODAY study demonstrated that metformin has minimal effects on weight reduction and is insufficient at maintaining HbA_1c_ over time [9], 10].

Glucagon-like-peptide 1 agonists (GLP1) are now widely recommended as both primary and add-on therapy for adults with type 2 diabetes, especially given their beneficial roles in weight reduction, as well as their cardiovascular and renal protective benefits [11]. In adults with type 2 diabetes, dulaglutide (1.5 and 0.75 mg) had greater efficacy compared to metformin in HbA_1c_ reduction (between group difference of 2.4 mmol/mol [0.22 %] and 1.6 mmol/mol [0.15 %]), respectively [12]. Weight loss effects were dose-dependent, with higher doses (3.0 and 4.5 mg) demonstrating greater weight reduction than the 1.5 mg dosage [13]. In adults with type 2 diabetes and obesity on background glucose-lowering medications, semaglutide injections (2.4 mg) over 68 weeks lowered HbA_1c_ by 13.1 mmol/mol (1.2 %) and reduced body weight by 6.2 % [14]. In treatment naïve adults with type 2 diabetes, semaglutide monotherapy up to 1.0 mg lowered HbA_1c_ by 16.4 mmol/mol (1.5 %) compared to placebo [15]. Compared to semaglutide, tirzepatide, a dual glucose-dependent insulinotropic polypeptide (GIP) and GLP1, demonstrated superior efficacy in decreasing both HbA_1c_ by 4.9 mmol/mol (0.45 %) and weight by 5.5 kg, establishing it as the most effective GLP1 for adults [16].

In recent years, several GLP1 have been approved for pediatric type 2 diabetes, and clinical practice guidelines support their use as an adjunctive treatment to metformin [8], 17]. Notably, while pediatric GLP1 randomized controlled trials (RCT) largely replicate the reduction in HbA_1c_ seen in adults, the doses approved in this population had marginal benefits in weight reduction [18], [19], [20]. Although higher doses of liraglutide (up to 3 mg) and semaglutide (up to 2.4 mg) have been shown to be effective in weight reduction in adolescents with obesity [21], 22], there is limited data on the efficacy of semaglutide and tirzepatide in youth with type 2 diabetes.

Presently, metformin remains the first-line recommendation in children with type 2 diabetes. The Treatment Options for Type 2 Diabetes in Adolescents and Youth (TODAY) and the Restoring Insulin Secretion (RISE) studies demonstrated that loss of beta cell function cannot be prevented by metformin monotherapy [10], 23]. The GLP1 doses currently approved for pediatric type 2 diabetes have limited to no efficacy in weight reduction. There is thus a pressing need to study the efficacy of the higher potency GLP1 in this population, as well as to determine their efficacy as monotherapy. Currently, GLP1 are recommended as add-on agents in pediatric type 2 diabetes. To our knowledge, there is no published data on the efficacy of GLP1 as a monotherapy at type 2 diabetes diagnosis in adolescents and young adults. This exploratory single-center study compares the real-world efficacy of metformin vs. GLP1 monotherapy on HbA_1c_ and BMI in newly diagnosed youth with type 2 diabetes.

## Materials and methods

**Figure 1:**
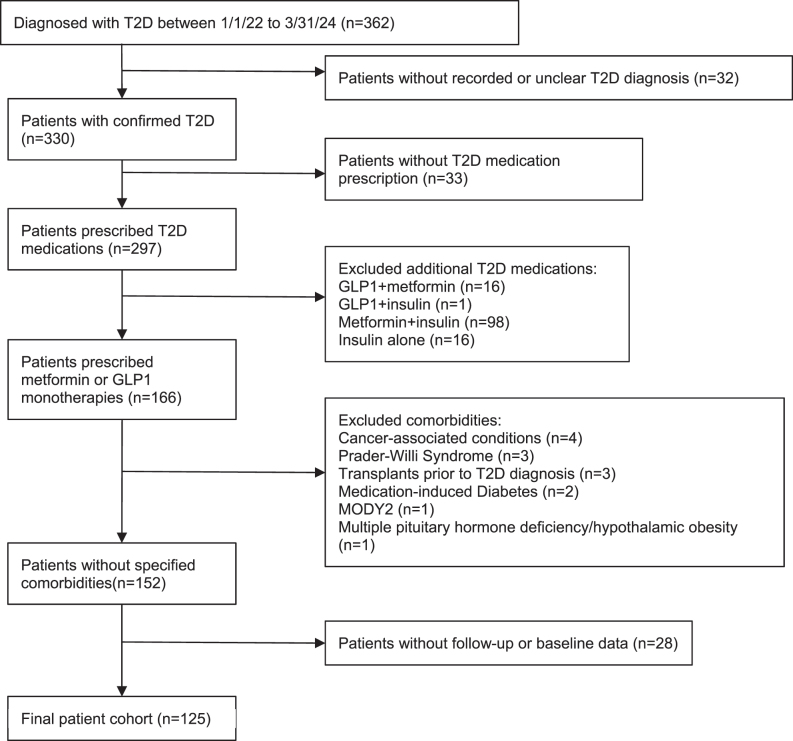
Flow diagram of study cohort.

Data were extracted from the electronic medical records of a large urban pediatric hospital. The study was approved by the Institutional Review Board (IRB) at Children’s Hospital Los Angeles. STROBE reporting guidelines for cohort studies were followed [24]. This study was conducted in accordance with the Declaration of Helsinki (as revised in 2013). Patient inclusion and exclusion criteria are detailed in Figure 1. The inclusion criteria included diagnosis with type 2 diabetes (based on provider diagnosis and retrieved from clinical database) between January 1, 2022 and March 31, 2024. Type 2 diagnosis is made in based on the American Diabetes Association (ADA) diagnostic criteria [8]. A total of 86 out of 125 patients had islet antibody results in the electronic medical records; two patients had GAD65 seropositivity, none was seropositive for IA-2 or insulin autoantibody. The time frame chosen aligned with the increased accessibility of GLP1 for adolescents with type 2 diabetes. Chart review was conducted to verify clinical documentation of the diagnosis of type 2 diabetes. The study period was limited to up to 1 year from the date of diagnosis. Additional inclusion criteria included prescription of metformin or a GLP1.

Exclusion criteria included type 2 diabetes management by lifestyle modification alone, initial medication regimen including insulin, or combination therapy (more than one antihyperglycemic medication). We also excluded other conditions with increased propensity for excess weight gain (Prader–Willi syndrome, hypothalamic obesity after brain tumor diagnosis), glucocorticoid-induced diabetes, post-transplant diabetes, leukemia, and monogenic diabetes. Patients with no baseline or follow-up HbA_1c_ and BMI z-score measure were also excluded from the study. Visits after bariatric surgery and the addition of a second antihyperglycemic or weight reducing medication were excluded from analysis as well.

Data collected included sex, age, race, ethnicity, insurance type, preferred language, duration of diabetes diagnosis, HbA_1c_, BMI, weight, height, and BMI z-score. The categorization of race and ethnicity is as previously described [25]. Records with missing, unknown, or “declined to state” were collapsed into the category of “non-conforming data” [26]. It should be noted that the term “non-conforming data” here is descriptive and non-normative; it simply reflects that those variables do not conform to the United State’ Office of Management and Budget’s race and ethnicity standards in either their original or expanded forms. For the GLP1 cohort, the final medication name, maximal dose prescribed, and adverse effects were recorded.

The primary outcome measures were changes in HbA_1c_ per month and percent change in BMI per month within a 1-year period from the initial diagnosis. Secondary outcomes included overall median changes and interquartile range (IQR) of both HbA_1c_, BMI, and BMI z-score within the same time frame. Summary statistics were used to describe variables in the dataset. Mann–Whitney *U* test was used for comparison of nonparametric variables. Linear regression models were fitted to assess the effect of medication on percent changes in BMI and HbA_1c_. Regression models were adjusted for gender, age, initial BMI, and initial HbA_1c_. Beta coefficients (β) derived from the model represented differences in changes of outcome measures for GLP1 compared to metformin. p-Values less than 0.05 were deemed as statistically significant. Chi-square test was used for the comparison of contingency analysis. All statistical analyses were performed using R Statistical Software (v4.2.3; R Core Team 2023).

## Results

### Patient demographic

From the initial records of 362 patients, 125 patients met inclusion and exclusion criteria and were used for analysis (Figure 1), including 12 patients in the GLP1 cohort and 113 patients in the metformin cohort. Patient demographics are described in Table 1. The median age of the entire cohort was 14.83 years [IQR 12.95, 16.58] and was comparable between the two groups (GLP1 14.93 [13.46, 17.32], metformin 14.83 [12.95, 16.44]). Compared to the metformin cohort, patients in the GLP1 group were mostly female (GLP1 83.3 %, metformin 51.3 %). More patients in the metformin group identified as Latino (GLP1 41.7 %, metformin 69.9 %). The two predominant languages spoken by patients were English and Spanish. Most patients were publicly insured. The median age and gender distribution of the overall study population is comparable to the general population of newly diagnosed pediatric T2D patients across the United States [27]. The race/ethnicity and insurance status are similar to previously reported for this pediatric diabetes center [28], 29]. The median duration of follow-up was comparable at 245 and 243 days, for the GLP1 and metformin groups, respectively.

**Table 1:** Baseline characteristics.

	Overall (n=125)	GLP1 (n=12)	Metformin (n=113)
Gender, n (%)			
Male	57 (45.6)	2 (16.7)	55 (48.7)
Female	68 (54.4)	10 (83.3)	60 (51.3)
Age, years [median, IQR]	14.83 [12.95, 16.58]	14.93 [13.46, 17.32]	14.83 [12.95, 16.44]
Race and ethnicity, n (%)			
Asian	3 (2.4 %)	1 (8.3 %)	2 (1.7 %)
Black	5 (4.0 %)	2 (16.7 %)	3 (2.7 %)
Hispanic or Latino	84 (67.2 %)	5 (41.7 %)	79 (69.9 %)
White	6 (4.8 %)	2 (16.7 %)	4 (3.5 %)
Nonconforming	27 (21.6 %)	2 (16.7 %)	25 (22.1 %)
Language, n (%)			
English	56 (44.8 %)	7 (58.3 %)	49 (43.4 %)
Spanish	66 (52.8 %)	5 (41.7 %)	61 (54.0 %)
Other	3 (2.4 %)	0 (0.0 %)	3 (2.7 %)
Insurance type, n (%)			
Public	119 (95.2 %)	12 (100 %)	107 (94.7 %)
Private	6 (4.8 %)	0 (0.0 %)	6 (5.3 %)
Diabetes duration, years, [median, IQR]	0.00 [0.00, 0.10]	0.00 [0.00, 0.03]	0.00 [0.00, 0.10]
Last visit day, [median, IQR]	243.00 [168.00, 322.00]	245.00 [187.75, 333.00]	243.00 [159.00, 315.00]

IQR, interquartile range.

### GLP1 dosage and side effects

GLP1 prescriptions changed over time based on medication availability. The final prescribed GLP1 and dosages are reported in Table 2. Once-weekly semaglutide 1 mg was the most prescribed (33.3 %) followed by tirzepatide 7.5 mg (25.0 %).

**Table 2:** Prescribed GLP1 and dosage.

Name (dose in mg)	n (%)
Dulaglutide	
1.5	1 (8.3)
3	1 (8.3)
Semaglutide	
0.5	1 (8.3)
1	4 (33.3)
2.4	1 (8.3)
Tirzepatide	
7.5	3 (25.0)
12.5	1 (8.3)

The side effect profile among GLP1 users was similar to prior studies [16], 18]. Seven (58.3 %) patients experienced at least 1 adverse effect throughout visits. With all visits combined (total of 23), the majority reported no adverse effects (13 visits, 56.5 %). The most common adverse effect was gastrointestinal (GI) issues, with reports of nausea, vomiting, diarrhea, and constipation (7 visits, 30.4 %). Other adverse effects include fatigue (1 visit, 4.34 %) and headache (2 visits, 8.70 %).

### Glycemic outcomes

Patients in both GLP1 and metformin groups had similar baseline and final HbA_1c_ levels (Table 3). Median HbA_1c_ for the GLP1 group decreased by 14.2 mmol/mol [IQR −16.4, −11.3] (baseline 52 mmol/mol, final 36 mmol/mol), compared to a reduction of 8.7 mmol/mol in the metformin group [IQR −16.4, −3.3]. Adjusted for duration of medication use, the monthly HbA_1c_ reduction was −1.7 mmol/mol and −1.1 mmol/mol for GLP1 and metformin groups, respectively. The difference was not statistically significant. In a model adjusting for gender, age, initial BMI z-score, and initial HbA_1c_, the change in HbA_1c_ per month favored GLP1 but was not statistically significant (β=−1.1%, p=0.308). We next examined the percentage of patients attaining the recommend HbA_1c_ target of 48 mmol/mol (6.5 %) for youth-onset type 2 diabetes [9,24]. At the end of the study period, 83 and 67 % of the patients in the GLP1 and metformin groups, respectively, attained an HbA_1c_≤48 mmol/mol (6.5 %) (p=0.253).

**Table 3:** Glycemic and BMI outcomes.

	Overall (n=125)	GLP1 (n=12)	Metformin (n=113)	p-Value
HbA1c				
Baseline, mmol/mol	52 [50, 65]	52 [50, 54]	52 [50, 67]	0.56
%	6.90 [6.70, 8.10]	6.90 [6.68, 7.12]	6.90 [6.70, 8.30]	
Final, mmol/mol	43 [37, 50]	36 [34, 43]	44 [37, 51]	0.03
%	6.10 [5.50, 6.70]	5.45 [5.27, 6.12]	6.20 [5.50, 6.80]	
Total change, mmol/mol	−9.8 [−16.4, −3.3]	−14.2 [−16.4, −11.3]	−8.7 [−16.4, −3.3]	0.11
%	−0.90 [−1.5, −0.30]	−1.30 [−1.5, −1.03]	−0.80 [−1.50, −0.30]	
Change per month, mmo/mol	−1.2 [−2.4, −0.4]	−1.7 [−2.5, −1.3]	−1.1 [−2.3, −0.4]	0.24
%	−0.11 [−0.22, −0.04]	−0.16 [−0.23, −0.12]	−0.10 [−0.21, −0.04]	
BMI, kg/m^2^				
Initial	36.00 [31.10, 42.20]	46.37 [35.50, 52.00]	35.06 [30.65, 41.30]	0.01
Final	34.97 [30.15, 41.50]	42.17 [33.98, 46.81]	34.64 [29.75, 41.25]	0.06
Total change	−0.54 [−1.66, 0.95]	−2.52 [−6.64, −1.81]	−0.18 [−1.33, 1.03]	<0.01
Change per month	−0.04 [−0.14, 0.09]	−0.43 [−0.60, −0.17]	−0.01 [−0.10, 0.10]	<0.01
BMI z-score				
Baseline	2.44 [2.06, 2.68]	2.61 [2.43, 2.81]	2.43 [2.03, 2.66]	
Final	2.32 [1.96, 2.63]	2.53 [2.20, 2.69]	2.30 [1.94, 2.63]	
Total change	−0.06 [−0.06, −0.06]	−0.12 [−0.23, −0.05]	−0.06 [−0.16, 0.01]	
Change per month	−0.01 [−0.02, 0.00]	−0.02 [−0.03, −0.01]	−0.01 [−0.02, 0.00]	

Median values reported; interquartile range reported in brackets.

### Weight reduction outcomes

**Figure 2:**
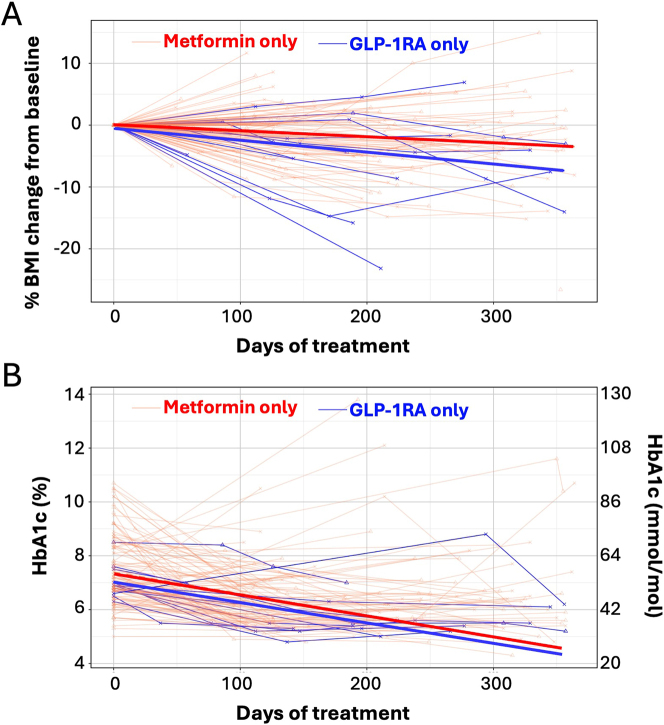
(A) Percent BMI change from baseline and (B) HbA1c in youth with type 2 diabetes treated with metformin vs. GLP1. Light lines – individual patient data. Bolded lines – best fit line from mixed effect linear regression model. Red lines – metformin. Blue lines – GLP1.

As shown in Table 3, baseline median BMI and z-scores were higher in the GLP1 group (BMI: GLP1 46.47 kg/m^2^ [35.50, 52.00], metformin 35.06 kg/m^2^ [30.65, 41.30]; BMI z-scores: GLP1 2.61 [2.43, 2.81], metformin 2.43 [2.03, 2.66]). At the end of the study, changes in median BMI favored the GLP1 group (GLP1 −2.52 kg/m2 [−6.64, −1.81], metformin −0.18 kg/m^2^ [−1.33, 1.03]). Change in BMI per month was also greater in the GLP1 group (GLP1 −0.43 kg/m^2^ [−0.60, −0.17], metformin −0.01 kg/m^2^ [−0.10, 0.10]). The change in median BMI z-score/month was −0.02 [−0.03, −0.01] and −0.01 [−0.02, 0.00] for GLP1 and metformin groups, respectively. Final percent BMI reduction favored the GLP1 group (GLP1 −5.10 % [−9.98, −3.79], metformin −0.59 % [−4.13, 1.72]; see Figure 2). In a regression model adjusting for gender, age, initial BMI, and initial HbA_1c_, GLP1 predicted greater reductions in percent BMI change/month (β= −1.08 %, p=0.001). In other words, patients prescribed GLP1 had an additional 1 % change in BMI per month, compared to those prescribed metformin.

## Discussion

This retrospective study compared the real-world efficacy of GLP1 monotherapy against metformin in youth with newly diagnosed type 2 diabetes in a single pediatric center. Over the study period of 12 months, the glycemic efficacy of GLP1 was similar to metformin. The percentage of patients reaching the recommended HbA_1c_ goal of 48 mmol/mol (6.5 %) was comparable between the two cohorts. GLP1 was more effective than metformin in reducing BMI, BMI z-score, and percent change in BMI. The side effect profile was similar to previously reported for medications in this class. To our knowledge, this observational study is the first to demonstrate the glycemic and weight reduction effects of GLP1 monotherapy compared to metformin in youth with newly diagnosed type 2 diabetes.

We present here the first head-to-head comparison of GLP1 against metformin in youth-onset type 2 diabetes in an observational study. The pivotal liraglutide trial showed an HbA_1c_ reduction of 7 mmol/mol (−0.64 %) from baseline, or −1.2 mmol/mol (−0.11 %) per month [20]. HbA_1c_ decreased by 8.5 mmol/mol (0.8 %) from baseline in the pooled dulaglutide arm (0.75 and 1.5 mg), or −1.4 mmol/mol (−0.13 %) per month [18]. Samuels et al. recently reported real world data that GLP1 usage predicted an average HbA_1c_ reduction of 1 mmol/mol (0.09 %) per month in two pediatric diabetes centers [30]. Our finding of median HbA_1c_ reduction of 1.7 mmol/mol (0.16 %) per month in the GLP1 group is comparable to previous reports. However, as metformin was the most prescribed background medication in the studies described above (76–88 %) [18], [19], [20, 30], the relative efficacy of GLP1 to metformin could not be estimated. In this exploratory study, our findings suggest that GLP1 may be comparable to metformin in its antihyperglycemic effect in newly diagnosed youth with type 2 diabetes. A larger GLP1 cohort may be needed to have sufficient power to detect differences in HbA_1c_ compared to metformin.

Most patients in this study attained the ADA recommended HbA_1c_ target of 48 mmol/mol (6.5 %) at the end of the study period (GLP1 83 %, Met 67 %). This finding seemingly contrasts with the general literature of rapid glycemic failure in youth-onset type 2 diabetes [6], 10]. We speculate that there are two reasons for this result. First, the relatively low HbA_1c_ at diagnosis suggests that this cohort likely has better beta cell function, which predicts durable glycemic control [31]. Second, the median last visit day (Table 3) was 243 days, or approximately 8 months, which is near the nadir of the HbA_1c_ progression after type 2 diabetes diagnosis [6]. A longitudinal study of greater duration is needed to determine the relative glycemic efficacy of GLP1 against metformin monotherapy.

Compared to metformin, weight reduction was superior in the GLP1 group, as measured by BMI, BMI z-score, and percent BMI change/month. This contrasts with the lack of weight loss effects in pivotal RCT of GLP1 in adolescents with type 2 diabetes [18], 20], 32]. We speculate that the most likely reason for the weight loss effect observed in this study is the choice and dosage of GLP1 used. In this study, most patients were prescribed GLP1 or GLP1/glucose-dependent insulinotropic polypeptide dual agonist considered to have very high efficacy for weight loss in adults (50 and 33.3 % were prescribed semaglutide and tirzepatide, respectively) [33]. At the time of this writing, no pediatric RCT data is currently available for semaglutide or tirzepatide in adolescents with type 2 diabetes. In adults with type 2 diabetes, however, Frias et al. have shown that all doses of tirzepatide are more efficacious than semaglutide 1 mg in its weight loss effect [16]. Our findings suggest that high efficacy GLP1 can effectively lower BMI in adolescents newly diagnosed with type 2 diabetes.

Rationale for considering GLP1 as monotherapy includes its beneficial effect on weight reduction, the simplicity of treatment schedule, and targeting the pathophysiology of youth-onset type 2 diabetes. In patients with class 2 or 3 obesity, metformin monotherapy is unlikely to attain clinically significant weight reduction, as recommended by current practice guidelines [8]. In the TODAY study, the BMI of those in the metformin arm increased by 0.4 ± 1.6 kg/m^2^ in the first 6 months after randomization [10]. Early initiation of high efficacy GLP1 may support weight reduction, mitigating the effect of obesity-related comorbidities. Weight loss has also been shown to predict durable glycemic control and reduce markers of cardiovascular disease [6], 7], 34]. For youth who are more motivated to use treatment for obesity than diabetes, this option may encourage adherence. Among adults with type 2 diabetes, fewer dosing periods is deemed one of the most favored attributes in discrete-choice experiments (DCE) [35]. Administering a once-weekly injection may be preferable to some patients compared to daily or twice-daily ingestion of metformin. Adolescents who are unable to swallow pills may also prefer weekly injections. GLP1 may also be introduced as an alternative for patients intolerant to the adverse side effects of metformin. Another reason to consider GLP1 as a monotherapy agent is its insulin sensitizing effect, which targets the insulin resistance of youth-onset type 2 diabetes. The SUSTAIN-1 study showed that semaglutide 1 mg in treatment naïve adults with T2D decreased HOMA-IR by 15 %, compared to placebo control [15]. The STEP TEENS phase 3a trial in adolescents with obesity also demonstrated a reduction in HOMA-IR over 30 % between participants receiving semaglutide 2.4 mg vs. placebo (−38.63 % vs. −5.84 %) at 68 weeks [36]. While the insulin-sensitizing effects GLP-1RAs are commonly attributed to weight loss [37], emerging evidence indicates a direct pharmacologic effect independent of weight reduction. Mashayekhi et al. recently demonstrated that liraglutide significantly improved markers of insulin sensitivity – including HOMA-IR, HOMA2, and the Matsuda index – after only 2 weeks of treatment, prior to any measurable weight loss [38]. GLP1’s beneficial effect on weight and insulin sensitivity as well as the reduced frequency of administration support the rationale for considering its usage as monotherapy in patients with new-onset type 2 diabetes.

The major strength of this study is that this is the first report on the efficacy of a head-to-head comparison of GLP1 against metformin in newly diagnosed adolescents with type 2 diabetes. We also minimized confounding effects on BMI and HbA_1c_ by excluding patients prescribed additional antidiabetes medications and data after bariatric surgery. Acknowledging that gender, baseline BMI, and HbA_1c_ may bias the choice of metformin vs. GLP1, a linear regression analysis was modeled to adjust for the effect of these variables on the primary outcome. Finally, we provide preliminary efficacy data on BMI and HbA_1c_ for semaglutide and tirzepatide, for which efficacy data are not yet available in adolescents with type 2 diabetes.

Inherent with retrospective studies, this study has several limitations. First, the higher baseline BMI in the GLP1 group implies prescribing bias, which may limit the generalizability of its efficacy to adolescents with lower BMI. In addition, the baseline HbA_1c_ in this study was approximately 11 mmol/mol (1 %) lower than those reported in the pediatric RCT of GLP1(18–20). The higher HbA_1c_ levels in RCT reflect a study population with a wide range of glycemic control, with some requiring metformin and insulin. This difference in baseline HbA_1c_ limits the generalizability of our findings to clinical populations that have higher HbA_1c_ levels. There was also an unequal distribution of gender, with female predominance in the GLP1 group. This skewed prescription pattern has been reported in adults as well [39], 40]. The increases in female prescription and usage may be explained by societal and gender pressures on weight management for women, which can persist from early childhood into adulthood. As a retrospective study, medication adherence was extracted from clinician documentation, which is limited by patient self-report. Under reporting of adherence may underestimate the effect size of GLP1 usage. The study duration is also constrained within the 1-year duration period after diagnosis, limiting its applicability on longer-term effects of GLP1 on HbA_1c_ and BMI z-score. Finally, the small sample size in the GLP1 group is another weakness of this exploratory study. The disparity in cohort sizes between GLP1 and Met groups reduces the accuracy of the comparisons, and any extrapolation from the results should be approached with caution. The difficulty in identifying a larger cohort reflects the general adherence to clinical practice guidelines of initiating metformin monotherapy at diagnosis. Our finding highlights the need to conduct RCT with sufficient power to validate our results, which has the potential to modify future clinical practice guidelines to consider GLP1 as a first-line medication for YT2D and obesity.

## Conclusions

In summary, this retrospective analysis of adolescents with newly diagnosed type 2 diabetes demonstrated that GLP1 is well tolerated and is more efficacious than metformin in weight reduction in the initial year after diagnosis. Future RCT are needed to validate this finding and to compare their effect on insulin sensitivity and beta cell function.
